# Isoniazid Induces Apoptosis Of Activated CD4^+^ T Cells: IMPLICATIONS FOR POST-THERAPY TUBERCULOSIS REACTIVATION AND REINFECTION*

**DOI:** 10.1074/jbc.C114.598946

**Published:** 2014-09-08

**Authors:** Sultan Tousif, Dhiraj Kumar Singh, Shaheer Ahmad, Prashini Moodley, Maitree Bhattacharyya, Luc Van Kaer, Gobardhan Das

**Affiliations:** From the ¶ Special Center for Molecular Medicine (SCMM), Jawaharlal Nehru University, ArunaAsaf Ali Marg, New Delhi 110067, India,; the ‡ School of Laboratory Medicine, College of Health Sciences, University of KwaZulu-Natal, Durban 4001, South Africa,; the § International Centre for Genetic Engineering and Biotechnology (ICGEB), ArunaAsaf Ali Marg, New Delhi 110067, India,; the ‖ Department of Biochemistry, University of Calcutta, 35, Ballygunge Circular Road, Kolkata 700 019, India, and; the ** Department of Pathology, Microbiology and Immunology, School of Medicine, Vanderbilt University, Nashville, Tennessee 37232

**Keywords:** Bacterial Pathogenesis, Cytokine, Immunology, Interleukin, T Helper Cells

## Abstract

Tuberculosis (TB) remains the second highest killer from a single infectious disease worldwide. Current therapy of TB is lengthy and consists of multiple expensive antibiotics, in a strategy referred to as Directly Observed Treatment, Short Course (DOTS). Although this therapy is effective, it has serious disadvantages. These therapeutic agents are toxic and are associated with the development of a variety of drug-resistant TB strains. Furthermore, patients treated with DOTS exhibit enhanced post-treatment susceptibility to TB reactivation and reinfection, suggesting therapy-related immune impairment. Here we show that Isoniazid (INH) treatment dramatically reduces *Mycobacterium tuberculosis* antigen-specific immune responses, induces apoptosis in activated CD4^+^ T cells, and renders treated animals vulnerable to TB reactivation and reinfection. Consequently, our findings suggest that TB treatment is associated with immune impairment.

## Teaser

**Background:** DOTS-treated patients exhibit vulnerability to reactivation and reinfection with TB, suggesting therapy-related immune impairment.

**Results:** Isoniazid (INH) dramatically reduces *Mycobacterium tuberculosis* antigen-specific immune responses by inducing apoptosis in activated CD4^+^ T cells.

**Conclusion:** Animals treated with INH exhibit post-treatment vulnerability to TB reinfection and reactivation.

**Significance:** The immune-impairing effects of antibiotics need to be considered in TB treatment.

## Introduction

*Mycobacterium tuberculosis*, the etiological agent of tuberculosis (TB),^4^ causes nearly 2 million deaths annually worldwide. One-third of the global population is infected with a latent form of TB, which represents an enormous reservoir awaiting an opportunity for reactivation (1). Conditions such as HIV infection that impair immunity may lead to TB reactivation (2). In the absence of HIV co-infection, only 5–10% of latently infected individuals develop TB in their lifetime, whereas 30% of co-infected individuals develop active TB (3). Consequently, a substantial number of deaths in HIV patients are associated with TB infection (4–6). Therefore, providers of TB treatment need to pay attention to restoring the immune status of the patient. Current internationally accepted therapy of TB by Directly Observed Treatment, Short Course (DOTS) consists of multiple expensive antibiotics and is lengthy (7, 8). The initial 2 months involve treatment with Isoniazid (INH), Rifampicin, Pyrazinamide, and Ethambutol, which is followed by an additional 4 months of treatment with INH and Rifampicin. Treatment of drug-resistant TB takes much longer (7–9). Because this treatment regimen involves multiple expensive antibiotics with substantial toxicity, a sizable number of patients withdraw from treatment, which is associated with the risk of generating drug-resistant TB variants (10, 11). Nearly all countries, irrespective of their socioeconomic conditions, are now under threat from drug-resistant TB (12, 13). Unfortunately, *M. tuberculosis* is acquiring drug resistance at a faster pace than the discovery of new generations of antibiotics, and this has already resulted in the appearance of a totally drug-resistant form of TB (14–16). Furthermore, these drugs are hepatotoxic (17). Importantly, after completion of DOTS, patients are more vulnerable to reactivation and reinfection of the disease, suggesting therapy-related immune impairment (18–20).

It is well established that CD4^+^ T cells play an important role in host resistance against TB (21). Additional studies have shown that impairments in IFN-γ production or mutations in the IFN-γ receptor are associated with TB susceptibility (22). Therefore, T helper (Th) 1 cells play a key role in host resistance (23). Nonetheless, recently, we and other groups of investigators have clearly demonstrated that Th1 responses are critically important for immune therapy and vaccine efficacy (24–26). Therefore, we considered it likely that treatment with antitubercular drugs impairs Th cell responses and that this causes increased susceptibility to post-treatment TB reactivation and reinfection. Among the antibiotics used during DOTS, INH and Rifampicin are used for the entire length of treatment. Further, INH is used for treatment of latent TB, but its potential effects on immune function remain incompletely understood. Because INH is a prodrug, *in vitro* studies of INH on immune cell functions are challenging. Therefore, in this study we have examined the *in vivo* effects of INH on immune responses in TB-infected animals.

Here we demonstrate that INH eliminates antigen-specific T cells by inducing apoptosis. Animals that are previously treated with INH exhibit increased susceptibility to TB reactivation and reinfection. In light of these findings, current therapeutic regimens for TB may need to be revised.

## MATERIALS AND METHODS

### 

#### 

##### Mice

Six- to 8-week-old BALB/c mice were used throughout our research. These mice were maintained at the institutional central animal facility. All animal experiments were conducted in accordance with the necessary guidelines of the Institutional Animals Ethics Committee. At different time points after infection with *M. tuberculosis*, all mice were humanely sacrificed by asphyxiation in carbon dioxide according to institutional and DBT regulations.

##### M. tuberculosis Low Dose Aerosol Infection

Infection with *M. tuberculosis* strain H37Rv (ATCC 27294; American Type Culture Collection, Manassas, VA) was performed using the low dose aerosol infection model. *M. tuberculosis* strain H37Rv was grown to mid-log phase (*A*_600_ ∼0.6) in Middlebrook 7H9 media (Difco^TM^) with 0.1% Tween 80 (Sigma), 0.2% glycerol, and 10% Middlebrook albumin, dextrose, and catalase enrichment medium (Difco^TM^). For future use, bacteria were kept at −80 °C in 20% glycerol stocks. Mice were infected with ∼150 cfu of *M. tuberculosis* H37Rv using an aerosol chamber. For aerosol infection, cultured stock was washed twice with PBS and made into a single cell suspension. Mice for infection studies were housed under barrier conditions in a BSL3 laboratory.

##### Drug Administration

Fifty mg/liter Isoniazid (Sigma) was administered *ad libitum* in drinking water.

##### Calculation of Colony-forming Units

Mice were sacrificed at the required time points, and organs were harvested, homogenized in 0.2 μm filtered PBS containing 0.05% Tween 80, and plated onto 7H11 Middlebrook (Difco^TM^) plates containing 10% oleic acid, albumin, dextrose, and catalase (Difco^TM^). Undiluted, 10-fold diluted, and 100-fold diluted homogenized lung and spleen cells were plated in duplicate on the 7H11 plates and incubated at 37 °C for 21 days. Colonies were counted, and cfu were calculated.

##### Antibodies and Reagents

We used the following antibodies and reagents: anti-CD4 (clone: GK1.5)-FITC, -PerCP-Cy5, or -APC, anti-CD8 (clone: 53-6.7)-FITC, -PerCP-Cy5, or -APC, anti-IFN-γ (clone: XMG1.2)-APC, anti-CD44 (clone: IM7)-APC, anti-BrdU (clone: Bu20a)-PE, 7-AAD, anti-IL12 (clone: C15.6)-PE, anti-IL4 (clone: 11B11)-PE, anti-IL22 (clone: Poly5164)-PE, anti-IL10 (clone: JES5-16E3)-PE, anti-IL9 (clone: MH9A4)-PE, and anti-CD69 (clone: H1.2F3)-PE (all from eBioscience); anti-caspase-3 (clone: C92605)-FITC (From BD Biosciences); anti-FasL (SC6237) (Santa Cruz Biotechnology); and IgG-Fab2 (clone: 4412) (Cell Signaling).

##### Flow Cytometry and Surface and Intracellular Staining

To prepare a single cell suspension, spleens and lungs were isolated from mice and macerated by frosted slides in 10% RPMI 1640 (Gibco, Life Technologies). Red blood cells (RBCs) were lysed with RBC cell lysis buffer, incubated at room temperature for 3–5 min, and washed with 10% RPMI 1640. The cells were counted, and 1 × 10^6^ cells were used for surface staining. For intracellular staining, 1 × 10^6^ cells were cultured per well in 24-well plates (Nunc) and activated with 50 ng/ml phorbol 12-myristate 13-acetate and 750 ng/ml ionomycin (Sigma) overnight, and 10 μg/ml brefeldin A (eBioscience) was added during the last 6 h of culture. Cells were washed twice with PBS and stained with antibodies directed against surface markers. After staining, cells were washed again with PBS, and cells were fixed with 100 μl of fixation buffer (eBioscience) for 30 min; the cells were then resuspended in 200 μl of permeabilization buffer (eBioscience) and stained with fluorescently labeled anti-cytokine antibodies. Fluorescence intensity of fluorochrome-labeled cells was measured by flow cytometry (FACSCanto^TM^ II, BD Biosciences). Cell viability dye (7-AAD) was added to the cells 15 min before analyzing the cells by flow cytometry. FACSDiva was used for acquiring the cells, and final data analysis was performed by FlowJo (Tree Star).

##### In Vitro T Cell Proliferation Assay

Spleens were isolated from infected or uninfected mice, macerated by frosted slides in 10% RPMI 1640 (Gibco, Life Technologies), and made into a single cell suspension. RBCs were lysed with RBC cell lysis buffer, incubated at room temperature for 3–5 min, and washed with 10% RPMI 1640. As described earlier (33), to isolate T lymphocytes, splenocytes were then passed through nylon wool columns. Similarly, lung T lymphocytes were isolated by filtration through nylon wool columns. T lymphocytes were counted and plated at 0.5 × 10^6^ cells/well in a 96-well plate or at 1 × 10^6^ cells/well in 24-well plates (Nunc) and stimulated with different concentrations of H37Rv bacterial protein lysate or *M. tuberculosis* complete soluble antigen (CSA). Cells were cultured for 48 h and then pulsed with tritiated thymidine (^3^H-labeled tritiated thymidine, 1.0 μCi/well; Amersham Biosciences) before measuring incorporation of ^3^H-labeled tritiated thymidine by means of a cell harvester and liquid scintillation counter 16 h later (Wallac TriLux, PerkinElmer).

##### In Vivo T Cell Proliferation Assay

Six hundred ng of BrdU in 100 μl of PBS was administered intraperitoneally to each mouse 3 days prior to the experiment. Cells from both lung tissue and spleen of mice were isolated and stained with anti-BrdU antibody in the presence of DNase. Data were acquired by flow cytometry.

##### Statistical Analysis

All data were analyzed by Excel 2007. In all figures, mean values were calculated with S.D. unless stated otherwise. For all statistical analyses, Student's *t* test was performed to compare two groups, and *p* < 0.05 was considered significant.

## RESULTS AND DISCUSSION

**FIGURE 1.**
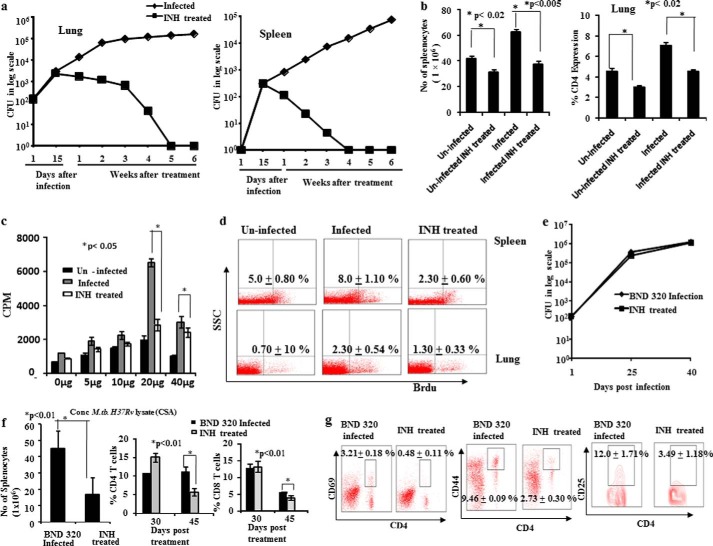
**The antituberculosis drug Isoniazid suppresses host immunity by inducing activation-induced cell death in T cells.** *a*, lung and spleen cfu. Mice infected with a high dose aerosol inoculum (∼150 cfu/mice) of *M. tuberculosis* strain H37Rv were sacrificed at various time points. Lungs and spleens were harvested, homogenized in 0.2 μm filtered PBS containing 0.05% Tween 80, and plated onto 7H11 Middlebrook plates containing 10% oleic acid, albumin, dextrose, and catalase. Undiluted, 10-fold, 100-fold, and 1000-fold diluted lung and spleen cell homogenates were plated in duplicate on the above 7H11 agar plates and incubated at 37 °C for 21 days. Colonies were counted, and cfu were estimated. Data shown here are representative of five independent experiments. Each cfu experiment has been carried out with three mice per experiment. *b*, *left panel*, total number of splenocytes 30 days post-treatment. Total numbers of splenocytes were counted using a hemocytometer after preparation of single cell suspensions following isolation of spleens from mice. Data shown here are representative of five independent experiments with three mice in each group and represent the mean ± S.D. values. *Right panel*, the percentage of CD4^+^ T cells in lungs. Lung lymphocytes were stained with anti-CD4 antibodies, and data were acquired by flow cytometry. The percentage of cells expressing CD4 is shown in the bar diagram with mean ± S.D. Data shown here are representative of five independent experiments with three mice in each group. *c*, antigen-specific T cell proliferation. T lymphocytes were isolated from spleens of mice 30 days post-treatment with INH, and T cell proliferation assays were performed using tritiated thymidine after stimulation with *M. tuberculosis* H37Rv CSA. Data shown here are representative of five independent experiments with three mice in each group and represent the mean ± S.D. values. *d*, *in vivo* T cell proliferation in spleen and lung. To determine the status of host T cell proliferation *in vivo* during infection and treatment, 0.6 mg of BrdU in 100 μl of PBS was administered intraperitoneally to each mouse at 3 days prior to sacrifice. Cells were then isolated from both lung tissue and spleen and stained with anti-BrdU antibodies. Data were acquired by flow cytometry. The percentage of cells showing BrdU incorporation in different groups at 30 days post-treatment is shown in the dot-plot diagram with mean ± S.D. Data shown here are representative of five independent experiments with three mice in each group. *e*, lung cfu. Mice infected with a high dose aerosol inoculum (∼150 cfu/mice) of the BND320 strain of *M. tuberculosis* were sacrificed at various time points, and lungs were harvested, homogenized in 0.2 μm filtered PBS containing 0.05% Tween 80, and plated onto 7H11 Middlebrook plates containing 10% oleic acid, albumin, dextrose, and catalase. Undiluted, 10-fold, 100-fold, and 1000-fold diluted lung cell homogenates were plated in duplicate on the above 7H11 plates and incubated at 37 °C for 21 days. Colonies were counted, and cfu were estimated. Data shown here are representative of three independent experiments. Each cfu experiment has been carried out with three mice per experiment. *f*, *left panel*, total number of splenocytes 45 days post-treatment. Total numbers of splenocytes were counted using a hemocytometer after preparation of single cell suspensions. Data shown here are representative of two independent experiments with three mice in each group and represent the mean ± S.D. values. *Middle* and *right panels*, the percentage of CD4^+^ and CD8^+^ T cells in spleen. Splenocytes were stained with anti-CD4 and anti-CD8 antibodies, and data were acquired by flow cytometry. The percentage of cells expressing CD4 or CD8 is shown in the bar diagram with mean ± S.D. at 30 and 45 days post-treatment. Data shown here are representative of three independent experiments with three mice in each group. *g*, CD69, CD44, and CD25 expression. Splenocytes of mice infected with BND320 and treated with INH for 45 days were surface-stained with anti-CD4, -CD69, -CD44, and -CD25 antibodies, and samples were acquired by flow cytometry. T cells were gated for CD4 expression, and the percentage of cells expressing CD69, CD44, and CD25 among CD4^+^ T cells is shown in the FACS plots with mean ± S.D. Data shown here are representative of three independent experiments with three mice in each group.

INH is a component of DOTS therapy and is employed for the entire length of treatment (7–9). Furthermore, INH monotherapy is used for treatment of latent TB (1, 9). Extended use of antitubercular drugs renders patients vulnerable to TB reactivation and reinfection, suggesting therapy-related immune impairment. Therefore, we determined the effects of INH on immune responses during the progression of TB disease in mice. We infected BALB/c mice with H37Rv and treated the animals with INH at a dose of 50 mg/liter. As expected, INH inhibited the growth of *M. tuberculosis* in a treatment time-dependent fashion (Fig. 1*a*). However, numbers of total splenocytes as well as activated CD4^+^ T cells declined rapidly (Fig. 1*b*). To assess whether INH treatment influences antigen-specific immune responses, we rechallenged splenocytes *ex vivo* with *M. tuberculosis*-derived CSA and measured cellular proliferation by ^3^[H]thymidine incorporation assay. We found that INH-treated animals exhibited a suppressed proliferative response as compared with untreated animals (Fig. 1*c*). This was further confirmed by using BrdU incorporation assays (Fig. 1*d*). However, we considered that each of these observed effects may be due to reduced availability of *M. tuberculosis* antigens in treated animals. To exclude this possibility, we performed similar experiments with an INH-resistant *M. tuberculosis* strain (BND320). Although we failed to observe any changes in bacillary growth induced by INH (Fig. 1*e*), treated animals exhibited reduced splenocyte numbers and CD4^+^ T cell proliferation, similar to that of the INH-sensitive strain H37Rv (Fig. 1, *f* and *g*). These findings indicated that INH suppresses antigen-specific immune responses.

**FIGURE 2.**
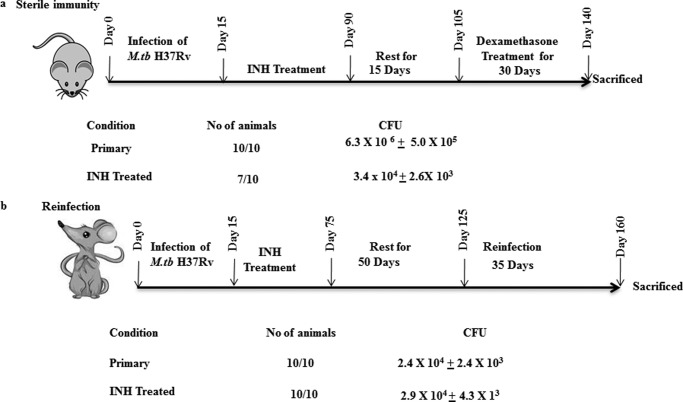
**INH is an inadequate inducer of sterile immunity and enhances the risk for reinfection.** *a*, treatment with Isoniazid induces poor sterilizing activity against latent TB. Mice infected with *M. tuberculosis* strain H37Rv following low dose aerosol exposure (∼150 cfu/mice) were treated with 50 mg/liter Isoniazid administered *ad libitum* (in the drinking water) until complete eradication of infection, *i.e.* for 75 days. These mice were then rested for 15 days followed by dexamethasone treatment (0.8 mg/kg administered intraperitoneally) three times per week for 30 days. Mice were then sacrificed, and cfu were analyzed from lung homogenates to estimate the reactivation of latent mycobacteria. *b*, Isoniazid treatment sensitizes animals to *M. tuberculosis* reinfection. Mice infected with *M. tuberculosis* strain H37Rv following the low dose aerosol infection model (∼150 cfu/mice) were treated with 50 mg/liter Isoniazid administered *ad libitum* until complete eradication of infection, *i.e.* for 60 days. These mice were then rested for 50 days followed by reinfection with *M. tuberculosis* using the same dose and protocol as for the primary infection. These mice were then sacrificed after 35 days, and lungs were harvested to evaluate the frequency of reinfection.

**FIGURE 3.**
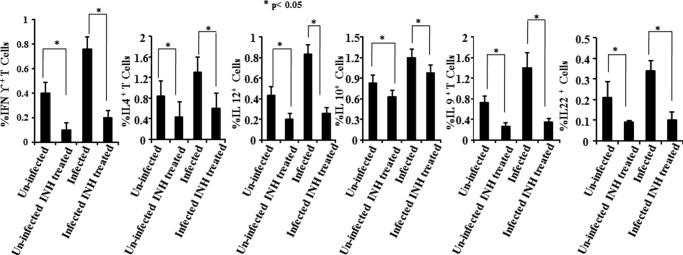
**INH suppresses cytokine production in splenocytes.** Splenocytes were cultured and activated with 50 ng/ml phorbol 12-myristate 13-acetate and 750 ng/ml ionomycin overnight, and 10 μg/ml brefeldin A was added during the last 6 h of culture. Cells were then intracellularly stained with anti-IL4, -IFNγ, -IL9, -IL10, -IL12, or -IL22 antibodies and appropriately labeled control antibodies. Cells were acquired by a flow cytometer. Data are shown as mean ± S.D., and Student's *t* test was performed for estimating significance between two groups.

We reasoned that INH might directly cause cell death in antigen-specific T cells. To investigate this hypothesis, we isolated spleen cells and lung T cells from infected and INH-treated animals and determined apoptosis in T cells. We found that a large number of cells stained with 7-AAD, indicating that INH induces apoptosis (data not shown). Cycling cells often have increased susceptibility to apoptotic death (27), and hence we determined whether T cell death was restricted to the activated T cell population. For this purpose, we stained the cells for expression of activation markers such as CD44 and CD69. As expected, we found that the loss of T cells was largely confined to the activated T cell population (data not shown). Apoptotic death is mediated by up-regulation of the caspase pathway, with caspase-3 playing a central role. Thus, we measured caspase-3 in apoptotic cells, and found it to be profoundly up-regulated (data not shown). To further strengthen this hypothesis, we examined the expression of FasL. We observed that FasL was dramatically up-regulated (data not shown). These observations indicated that, upon treatment with INH, activated CD4^+^ T cells undergo apoptosis. To find out the most probable reasons for vulnerabilities of reactivation and reinfection (Fig 2) after treatment of tuberculosis infection, we checked the status of Th1 cells in INH-treated mice. Because it is well known that Th1 cells are more susceptible to apoptosis than Th2 cells (28), we tested whether INH has a biased activity in inducing apoptosis in Th1 *versus* Th2 cells. For this purpose, we measured cytokines in the splenocytes. We observed that Th1 cells underwent apoptosis more rapidly than Th2 cells (Fig. 3). Th1 cells (producers of IFN-γ) play a central role in host protection against TB, and *M. tuberculosis* thwarts such responses by several means for its unhindered lifestyle within susceptible hosts (29). Therefore, elimination of Th1 cells by INH may be detrimental and render the host susceptible to reactivation or reinfection after treatment. In this context, the available literature indicates that patients treated with DOTS therapy are more vulnerable to disease reactivation and reinfection after completion of the therapeutic regimen (18, 19, 20). Therefore, these studies suggest that therapeutic antibiotics influence antitubercular immune responses. As susceptibility to *M. tuberculosis* infection is determined by Th cells (30), we reasoned that loss of CD4^+^ T cells during treatment may play a role in post-treatment vulnerability to TB reactivation and reinfection. To test this hypothesis, we treated animals with a course of INH and subsequently either treated the animals with dexamethasone or reinfected them with *M. tuberculosis* H37Rv. As shown in Fig. 2*a*, treatment with dexamethasone promoted *M. tuberculosis* reactivation in INH-treated animals, as deduced from the bacterial counts in different organs. This observation suggested that primary treatment resulted in the killing of most *M. tuberculosis* organisms, but did not induce complete eradication. Therefore, an alteration in the status of the immune response reactivates the latent organisms. It is interesting to note that INH treatment induces apoptosis in activated Th cells, and although INH is unable to completely eliminate the *M. tuberculosis* organisms, the organisms do not immediately reactivate after treatment is completed. It is highly likely that *M. tuberculosis* hides in secluded structures such as granulomas embedded in an immunosuppressive environment, where drug penetration is low. Previously, we have shown that mesenchymal stem cells contribute to this immunosuppressive environment (31), and other host cells may contribute as well (32).

Recently, we and other groups of investigators have shown that Th1 cells play an important role in vaccine efficacy against tuberculosis (24–26). Here we have presented data indicating that INH partially eliminates *M. tuberculosis* antigen-specific CD4^+^ T cells. This may render treated animals more susceptible to TB reinfection. As shown in Fig. 2*b*, we found that animals previously treated with INH were as susceptible as naive animals to TB infection. This observation suggested that, despite the generation of host protective immunity during infection and clearance of the organisms, immune memory has not been restored. It is likely that the activated CD4^+^ T cells eliminated by INH are precursors to the central memory cells that are critical for eliciting vaccine efficacy.

Therefore, our observations warrant consideration for the addition of immunomodulators to conventional antibiotic treatment regimens. Such immunomodulators may prevent the loss of antigen-specific T cells during treatment. Thus, restoration of immunological memory in this manner may act as a self-propagating vaccine to protect against TB reactivation and future infection.

## Footnotes

4 The abbreviations used are:

TB: tuberculosis
DOTS: Directly Observed Treatment, Short Course
INH: Isoniazid
Th: T helper
CSA: complete soluble antigen
7-AAD: 7-aminoactinomycin D
APC: allophycocyanin
PE: phycoerythrin.
